# An environmental study of tracheostomy on eight COVID-19 patients

**DOI:** 10.1186/s40463-021-00494-1

**Published:** 2021-01-18

**Authors:** Kai Xu, Xin-Hao Zhang, Xiao-Bo Long, Xiang Lu, Zheng Liu

**Affiliations:** Department of Otorhinolaryngology Head and Neck Surgery, Tongji Hospital, Tongji Medical College, Huazhong University of Science and Technology, Wuhan, 430030 China

**Keywords:** COVID-19, SARS-CoV-2, Tracheostomy, Viral shedding

## Abstract

**Background:**

Tracheostomy, as an aerosol-generating procedure, is considered as a high-risk surgery for health care workers (HCWs) during the coronavirus disease (COVID-19) pandemic. Current recommendations are to perform tracheostomy after a period of intubation of > 14 days, with two consecutive negative throat swab tests, to lower the risk of contamination to HCWs. However, specific data for this recommendation are lacking. Therefore, this study aimed to evaluate viral shedding into the environment, including HCWs, associated with bedside tracheostomy in the intensive care unit.

**Methods:**

Samples obtained from the medical environment immediately after tracheostomy, including those from 19 surfaces, two air samples at 10 and 50 cm from the surgical site, and from the personal protective equipment (PPE) of the surgeon and assistant, were tested for the presence of severe acute respiratory syndrome coronavirus 2 in eight cases of bedside tracheostomy. We evaluated the rate of positive tests from the different samples obtained.

**Results:**

Positive samples were identified in only one of the eight cases. These were obtained for the air sample at 10 cm and from the bed handrail and urine bag. There were no positive test results from the PPE samples. The patient with positive samples had undergone early tracheostomy, at 9 days after intubation, due to a comorbidity.

**Conclusions:**

Our preliminary results indicate that delayed tracheostomy, after an extended period of endotracheal intubation, might be a considerably less contagious procedure than early tracheostomy (defined as < 14 days after intubation).

## Background

As of January 1, 2021, over 80 million cases of coronavirus disease (COVID-19) have been reported globally, severely burdening health care systems. The severe acute respiratory syndrome coronavirus 2 (SARS- CoV-2) has been identified as the cause of this highly contagious disease [1]. The main transmission routes for SARS-CoV-2 are respiratory droplets and close contact [2]. Whether SARS-CoV-2 can be transmitted by aerosols, however, remains controversial due to inconsistent evidence [3–6].

The primary morbidity associated with COVID-19 is acute respiratory distress syndrome, which may require invasive mechanical ventilation, including tracheostomy [7]. Tracheostomy plays a crucial role in weaning from mechanical ventilation and in improving patient care for better outcomes. However, as an aerosol-generating procedure, tracheostomy is usually associated with high droplet and particle generation, which has been considered as one of the highest contamination risks for health care workers (HCWs) during the COVID-19 pandemic [8, 9]. Knowing the possible extent of environmental contamination of SARS-CoV-2 during tracheostomy in units treating COVID-19 patients is critical for improving safety practices for HCWs. This information could also guide decision-making regarding the indications and appropriate timing for tracheostomy and the strategy that would mitigate the risk for nosocomial infections, while maintaining the quality of patient care.

Within the current context of the SARS-CoV-2 pandemic, it has been suggested that tracheostomy be performed once a patient’s viral load has declined to a negligible level, which requires extending the period of endotracheal intubation for ≥2–3 weeks before tracheostomy [10]. However, there is a lack of direct supporting evidence regarding the safety of this suggestion with regard to the risk for infection. To address this current gap in practical knowledge, we evaluated the risk of SARS-CoV-2 contamination in samples collected from HCWs and surfaces in proximity of patients who underwent bedside tracheostomy in the intensive care unit.

## Methods

### Statement of ethics

Our study design was approved by our Institutional Review Board, and written informed consent was obtained from the patients’ representatives.

### Study design and group

This was a descriptive case series on viral shedding during tracheostomy. Between March 3 and April 4, 2020, eight patients with laboratory-confirmed SARS-CoV-2 infection underwent tracheostomy at bedside at the designated COVID-19 treatment center for critically ill patients at Wuhan Tongji Hospital.

### Sample collection

For each patient, we obtained samples at 21 sites immediately after tracheostomy: 19 samples from surfaces and two samples from the air in the patients’ proximity. We also obtained samples from the personal protective equipment (PPE) worn by the surgeon and the assistant immediately after tracheostomy. The timings of tracheostomy and the procedure were consistent with current recommendations [10, 11]. All patients had been under medical care at the hospital for a number of weeks and had undergone frequent SARS-CoV-2 testing using throat swabs. The decision to proceed with tracheostomy was made after two consecutive negative test results. Following previously described medthods [3, 12], samples from surfaces were acquired using sterile premoistened swabs. Air samples were collected from the start of the tracheostomy procedure using a MicroBio MB2-RSH Isolator Microbial Air Sampler (Cantium Scietntific, Kent, UK) with gelatin membrane filter. Air sampling was performed for 50 min, at a rate of 50 L/min, and at distances of 10 cm and 50 cm from the surgical site.

### Tracheostomy procedure

Due to the current lack of evidence regarding the safe timing of tracheostomy for COVID-19 patients, we used the highest level of precaution during the surgery [8, 13, 14], which included using the following PPE: waterproof medical cap, N95 medical respirator, protective garment, anti-penetration isolation gown, two layers of surgical gloves, plastic shoe covers, and powered air-purifying respirators (PAPRs) [15]. Tracheostomy was performed according to recently published protocols [8, 11, 16]. Briefly, patients were pre-oxygenated and fully sedated. Three HCWs participated in the surgery, including a surgeon, a surgical assistant, as well as an anesthesia specialist for managing the endotracheal tube. Following routine open tracheostomy, mechanical ventilation was terminated as the surgeon was ready to perform the tracheal incision. No cautery or suction was used during the surgery. The endotracheal tube was then pulled out, and the tracheostomy tube was inserted, followed by inflation of the balloon cuff.

### Analysis of samples

Specific real-time reverse transcriptase polymerase chain reaction (RT-PCR), targeting RNA-dependent RNA polymerase, was used to detect the presence of SARS-CoV-2 RNA. Samples were collected and stored in a collection tube with 5 mL virus preservation solution. RNA was isolated using the Tianlong PANA9600 automatic nucleic acid extraction system (Tianlong, Xi’an, China). The open reading frame 1ab (ORFlab) and nucleocapsid protein (NP) genes were simultaneously tested using a commercial RT-PCR kit from DAAN GENE (Guangzhou, China). RT-PCR was performed using the Tianlong Gentier 96E real-time PCR system under the following conditions: 50 °C for 15 min; 95 °C for 15 min, 45 cycles of 94 °C for 15 s, and 55 °C for 45 s for fluorescence measurement.

A cutoff cycle threshold (Ct) value of 40 was used for both genes, with a value < 40 defined as a positive test, and lower Ct values indicating of a higher viral load. Relevant clinical data (age, initial symptoms, number of days since intubation and since symptom onset, and RT-PCR results) were collected for analysis.

## Results

Table 1 summarizes the patient and clinical data and sample test results. With the exception of patient 7 in Table 1, all patients underwent tracheostomy ≥14 days after endotracheal intubation. Patient 7 was diagnosed with cerebral hemorrhage at the time of intubation and underwent tracheostomy 9 days after endotracheal intubation, and 33 days after COVID-19 symptom onset. Four repeated throat swabs obtained before tracheostomy showed no viral shedding.

**Table 1:** Patients’ basic information and sampling results

Patient	Age	Days since intubation	Days since symptom	Initial symptoms	Comorbidities	Indications for tracheotomy	Outcome	Air positive samples	Object surface positive samples
1	55	27	40	Fever, cough	Hypertension, diabetes	Prolonged incubation	Deceased	**0/2**	**0/19**
2	70	27	37	Fever	Cerebral infarction, hypertension, diabetes	Prolonged incubation	Deceased	**0/2**	**0/19**
3	65	21	34	Fever, cough	Cerebral hemorrhage, hypertension, diabetes	Prolonged incubation	Partial recovery	**0/2**	**0/19**
4	67	17	24	Fever, cough, fatigue	Cerebral hemorrhage, hypertension, diabetes	Prolonged incubation	Deceased	**0/2**	**0/19**
5	69	24	46	Fever, cough	Hypertension, diabetes	Prolonged incubation	Deceased	**0/2**	**0/19**
6	69	36	47	Fever, fatigue	Cerebral infarction	Prolonged incubation	Partial recovery	**0/2**	**0/19**
7	68	9	33	Cough, fatigue	Cerebral hemorrhage, hypertension, diabetes	Failure to wean	Deceased	**1/2**	**2/19**
8	74	27	56	Fever, cough	Hypertension, acute cardiac injury	Prolonged incubation	Deceased	**0/2**	**0/19**

All samples were negative for SARS-CoV-2, except samples obtained for patient 7. The following samples tested positive for this patient: air sample at a distance of 10 cm from the surgical site and surface samples from the bed handrail and the urine bag (Table 2). All PPE swabs were negative. All air samples at a distance of 50 cm from the surgical site were negative.

**Table 2:** Environmental and PPE Sites Sampled and Corresponding RT-PCR Results

Site	Positive samples (patient 7)	Cycle threshold value
Environmental sites		
Air (10 cm away from the surgical site)	1/8	38.2
Air (50 cm away from the surgical site)	0/8	
Floor (50 cm away from the surgical site)	0/8	
Quilt (50 cm away from the surgical site)	0/8	
Air outlet fan	0/8	
Monitor	0/8	
Sickbed handrail	1/8	36.8
Urine bag	1/8	37.5
Airway secretion	0/8	
Injection pump	0/8	
Ventilator screen	0/8	
Bedside surgical lamp	0/8	
water in ventilator circuit	0/8	
Staff PPE sites		
Surgeon’s PAPR	0/8	
Surgeon’s gloves	0/8	
Surgeon’s gown	0/8	
Surgeon’s front of shoes	0/8	
Assistant’s PAPR	0/8	
Assistant’s gloves	0/8	
Assistant’s gown	0/8	
Assistant’s front of shoes	0/8	

All nine HCWs involved in tracheostomy procedures underwent following routine examination 2 weeks after their medical rotations: routine blood test, chest computed tomography, SARS-CoV-2 testing using throat swabs, and serum titer of SARS-CoV-2 antibodies. None of the individuals in the tracheostomy medical group showed a positive result for COVID-19 infection.

## Discussion

One major concern for HCWs managing COVID-19 patients on prolonged endotracheal intubation is the risk of viral exposure during tracheostomy. Beside the risk of potential transmission of SARS-CoV-2 droplets, viral aerosols generated in the airway and ventilator circuit during the surgery should not be neglected [17]. Respiratory secretions are known to be aerosolized through daily activities (e.g., exhaling and coughing) and medical procedures (e.g., endotracheal intubation, bronchoscopy, and tracheotomy) [14, 17]. A recent study reported that viable SARS-CoV-2 has been cultured in the air in hospital wards with COVID-19 patients [12]. Thus, retesting for viral shedding status prior to proceeding with tracheostomy was recommended by multiple protocols [14, 18]. In this study, we provided the direct supporting evidence that patients who underwent delayed tracheostomy (≥14 days after tracheal intubation) showed no detectable SARS-CoV-2 RNA in the surgical environment, a finding consistent with the negative results of viral shedding from their throat swabs. The only patient who showed potential transmission risk was the one who underwent tracheostomy only 9 days after endotracheal intubation. As previously described, patient 7 was diagnosed with cerebral hemorrhage at the time of intubation, resulting in a loss of consciousness which prevented successful weaning from the ventilator over the next several weeks. Considering the onset of symptoms for this patient at 33 days previously and an average period of intubation of 5 days before symptom onset [2], at the time of tracheostomy, this patient had theoretically exceeded the most prolonged period of viral shedding documented to date [19, 20]. Accordingly, after a discussion with the consulting physician, we performed the procedure to improve patient outcomes.

Considering that tracheostomy is an aerosol and droplet generating procedure, the positive air samples we collected from patient 7 could have resulted from either of these transmission routes. During the procedure, the trachea was exposed to air for only 1–2 min; however, this does not exclude the possibility of droplets splashing over the 10-cm distance from the surgical site.

For patient 7, four repeated negative throat swabs were obtained over the nine days of intubation, with two subsequent swabs obtained after the tracheostomy also being negative. A study from Singapore reported a higher positive rate of SARS-CoV-2 RNA in samples from bronchoalveolar lavage fluid (93%) compared with throat swabs (32%) [21]. In our study, samples via a direct swab of secretions on the extubated endotracheal tube and a swab from water in the ventilator circuit, both tested negative for SARS-CoV-2 RNA. The positive air sample from patient 7 suggests that sputum from lower bronchial regions would better reflect the viral shedding status during tracheostomy in COVID-19 patients.

The positive sample result from the handrail of the bed for patient 7 is consistent with that of a previous study that reported 6 positive samples out of 14 from handrails of beds, which was among the highest rate of positivity for any surface in the proximity of a patient with COVID-19 [3]. To date, no data have been reported on SARS-CoV-2 viral shedding through urine [21]. Thus, our positive sample result from the urine bag of patient 7 may possibly be because of cross-contamination from other sites or another patient.

All air samples obtained from a distance of 50 cm from the surgical site, which mimics the distance between HCWs and the surgical site during tracheostomy, were negative. All PPE swabs also tested negative for viral shedding. These results indicate that delayed tracheostomy has a relatively lower risk of infection than endotracheal intubation. In our study, because of the high precautions taken, such as the use of PAPRs, and the improved surgical workflow, which limited aerosol exposure, contributed to the absence of COVID-19 infection among the HCWs in our group.

The limitations of our study need to be acknowledged. First, our case series included only eight patients, a relatively small sample, from one center in Wuhan, China. Further recruitment, within the time frame of our study, was not possible due to the resolution rate of the COVID-19 outbreak in Wuhan. Second, because the air samples were taken at 10 and 50 cm distances simultaneously, the airflow disturbance between them may cause bias. Third, positive samples for SARS-CoV-2 were identified in only one of the eight cases included, and the cycle threshold values were “weakly” positive which can likely be attributed to residual viral shedding [22]. Lastly, the false-negative rates of RT-PCR with simultaneous ORFlab and NP genes testing range from 11% ~ 49% [23]. For patients who had been infected by SARS-CoV-2 more than three weeks, the probability of a false-negative RT-PCR result was over 60% [24]. Meanwhile, the positive results of the nucleic acid test did not indicate the existance of viable intact virus, as well as the risk of transmission. Thus, we could not determine the transmission potential.

## Conclusion

In conclusion, the results of our preliminary results indicate that delayed tracheostomy, after an extended period of endotracheal intubation, showed no detectable SARS-CoV-2 RNA in the surgical environment. This might make it a considerably less contagious procedure than early tracheostomy (defined as < 14 days after intubation) for COVID-19 patients. Multicenter collaborations with a larger sample size are required to address remaining questions regarding the role and relevance of bronchoalveolar lavage or lower airway sampling in the safety of performing tracheostomy on COVID-19 pandemic.

## Data Availability

The datasets used and/or analyzed during the current study are available from the corresponding author on reasonable request.

## Abbreviations

HCWs: Health care workers
COVID-19: Coronavirus disease 2019
SARS- CoV-2: Severe acute respiratory syndrome coronavirus 2
PPE: Personal protective equipment
PAPRs: Powered air-purifying respirators
RT-PCR: Reverse transcriptase–polymerase chain reaction
